# Correlation of blood–brain barrier leakage with cerebral small vessel disease including cerebral microbleeds in Alzheimer's disease

**DOI:** 10.3389/fneur.2023.1077860

**Published:** 2023-02-16

**Authors:** Zhaozhao Cheng, Linbin Dai, Yan Wu, Yuqin Cao, Xianliang Chai, Peng Wang, Chang Liu, Ming Ni, Feng Gao, Qiong Wang, Xinyi Lv

**Affiliations:** ^1^Department of Neurology, The First Affiliated Hospital of USTC, Division of Life Sciences and Medicine, University of Science and Technology of China, Hefei, China; ^2^Neurodegenerative Disorder Research Center, Division of Life Sciences and Medicine, University of Science and Technology of China, Hefei, China; ^3^Department of Radiology, The First Affiliated Hospital of USTC, Division of Life Sciences and Medicine, University of Science and Technology of China, Hefei, China; ^4^Department of Nuclear Medicine, The First Affiliated Hospital of USTC, Division of Life Sciences and Medicine, University of Science and Technology of China, Hefei, China

**Keywords:** Alzheimer's disease, amyloid -beta, cerebral small vascular disease (CSVD), cerebral micro bleeds, blood–brain barrier

## Abstract

**Background:**

Blood–brain barrier (BBB) damage is considered an important part of Alzheimer's disease (AD) progression, and cerebral small-vessel disease (CSVD) is commonly associated with AD. However, the relationship between BBB damage, small cerebrovascular lesions, especially cerebral microbleeds (CMBs), and amyloid and tau biomarkers remains controversial. Therefore, our study aimed to further investigate their association in our cohort of patients with AD.

**Methods:**

A total of 139 individuals were divided into probable AD (^18^F-florbetapir PET positive, *n* = 101) and control group (cognitively normal, *n* = 38). The levels of cerebrospinal fluid (CSF) and plasma t-tau, p-tau181, Aβ40, Aβ42, and albumin were measured using corresponding commercial assay kits, and the CSF/plasma albumin ratio (Qalb), an indicator of BBB dysfunction, was calculated. CSVD burden and the number of CMBs were defined using magnetic resonance imaging.

**Results:**

Patients with AD had higher Qalb (*p* = 0.0024), higher numbers of CMBs (*p* = 0.03), and greater CSVD burden (*p* < 0.0001). In the AD group, CMBs and CSVD correlated with a higher Qalb (*p* = 0.03), and the numbers of CMBs negatively correlated with CSF Aβ42 (*p* = 0.02).

**Conclusion:**

Blood–brain barrier damage was accompanied by a more severe burden of CSVD, including CMB, in patients with AD.

## Introduction

Alzheimer's disease (AD) is a complex neurodegenerative disorder characterized by memory loss and cognitive impairment (1). The formation of neuronal amyloid-β (Aβ) plaques and tau protein hyperphosphorylation are histopathologic characteristics of AD (2–5). Many factors are involved in the development of AD, including age, heredity, obesity, diabetes, hypertension, and brain trauma (6–8). Recently, early blood–brain barrier (BBB) breakdown that contributes to AD pathogenesis has been increasingly recognized (9). The BBB, which is an important boundary between the blood and brain, is mainly formed by vascular endothelial cells of the central nervous system (CNS) and relies on continuous complexes of tight junctions (9–12). CSF/plasma albumin quotient (Qalb) is an ideal measure for the permeability of the BBB. Because albumin is only synthesized in the liver, albumin in the cerebrospinal fluid is therefore derived only from the blood and is transported to the central nervous system by passive diffusion (13). Thus, an abnormal elevation of Qalb is thought to reflect increased BBB permeability. In previous studies, Musaeus et al. similarly found that Qalb can contribute to the identification of different types of dementia, which may also be related to the impairment of BBB (14). BBB damage and vascular dysregulation can be detected before a cognitive decline in patients with AD (15, 16). Meanwhile, a large number of studies also indicated that Aβ accumulation is generally accompanied by the occurrence of cerebral small-vessel disease (CSVD) (16), indicating that there is a synergistic effect between BBB damage and CSVD in the occurrence of AD. CSVD involves a series of pathological processes, including perforation of the cerebral arterioles, venules, and capillaries (17). White matter hyperintensity (WMH) and cerebral microbleeds (CMBs), identified using magnetic resonance imaging (MRI), are diagnostic markers of CSVD (18).

Previous studies evaluating the relationship between brain Aβ (using PET imaging), CSVD, and cognition have shown conflicting results. Some studies found no relationship between brain Aβ and WMH in individuals with cognitively normal, mild cognitive impairment (MCI), subcortical vascular MCI, and AD (19), while a few studies reported that higher brain Aβ correlated with higher WMH volumes (20, 21). Another study using PET to show Aβ compliance found no interaction effect between Aβ and lacunae or CMB in AD (22). The pathogenesis of CMB includes small vascular wall damage caused by vascular risk factors and the accumulation of Aβ. Therefore, the development of cerebral microhemorrhage in patients with AD may help explain the overlap of cerebrovascular and neurodegenerative pathological mechanisms in cognitive dysfunction and dementia (23). Moreover, whether microvascular disease damage is associated with BBB damage in AD remains unclear.

In this study, we investigated the association between CMB and the BBB dysfunction measure, Qalb, as well as fluid biomarkers of Ab/tau in patients with AD. Our findings may deepen our understanding of the impact of small cerebrovascular lesions and BBB breakdown on AD pathology.

## Materials and methods

### Participants

Individuals, including patients with AD and cognitively normal (CN) controls, were selected from the China Aging and Neurodegenerative Disorder Initiative (24) cohort in the First Affiliated Hospital of the University of Science and Technology of China (USTC) from 2018 to 2022. All patients were diagnosed in accordance with the diagnostic guidelines based on the 2011 NIA-AA of probable AD and further underwent the Mini-Mental State Examination (MMSE) (25). The exclusion criteria were other types of dementia, such as frontotemporal dementia, Lewy body dementia, vascular dementia, Parkinson's disease dementia, and other brain disorders such as stroke, brain cancer, intellectual disability, severe depression, and/or severe weakness, leading to invalid mental health assessment. All patients with AD were visually screened for positivity by ^18^F-florbetapir PET by two experienced physicians as described in previous reports (26). Meanwhile, normal cognition volunteers with no history of dementia or other neuropathological abnormalities were recruited as the control group. Written informed consent was obtained from each patient, in accordance with the Declaration of Helsinki. The protocols used in this study were reviewed and approved by the ethical committee of the First Affiliated Hospital of USTC (2019KY-26).

### Sample collection and fluid biomarkers measurement

Cerebrospinal fluid (CSF) and plasma samples were collected in the morning after overnight fasting. After centrifugation, the samples were divided into 200 μL aliquots in polypropylene tubes and stored at −80°C until measurements were taken. CSF and plasma Aβ40, Aβ42, P-tau181, and total tau were measured on the HD-X analyzer (Quanterix) using Simoa kits (Quanterix, 103714, 101195). CSF albumin levels were measured using commercial assay kits (Thermo Fisher Scientific). Serum albumin levels were determined using a bromocresol green dye-binding assay (ADVIA 1800; Siemens, Berlin, Germany). The Qalb value was calculated using the following formula: (CSF albumin/serum albumin) × 1,000.

### APOE genotyping

Genomic DNA was purified from 200 μL of whole blood using the EasyPure Blood Genomic DNA kit (TransGen Biotech, EE121-11) according to the manufacturer's instructions. Genomic DNA was then used to amplify a 268-bp DNA fragment using the primers F-5′GGCACGGCTGTCCAAGGA and R- 5′CTCGCGGATGGCGCTGAG. Next, HhaI (NEB, R0139S) was added to the mixture of the PCR products for restriction enzyme digestion. Finally, the reaction mixture was loaded onto a 12% polyacrylamide non-denaturing gel and electrophoresed for 3 h under constant current–voltage (80 V). The APOE gene type was determined according to the size of HhaI fragments visualized by UV transillumination following the unique digestion pattern: APOE2/2 (91 and 83 bp); APOE2/3 (91, 83, 48, and 35 bp); APOE2/4(91, 83, 72, 48, and 35 bp); APOE3/3 (91, 48, and 35 bp); APOE3/4 (91, 72, 48, and 35 bp), and APOE4/4 (72, 48, and 35 bp).

### Neuroimaging marker assessments

All MRIs, including T1, T2, FLAIR, and SWI, were assessed by two experienced neuroradiologists blinded to clinical information. Based on the recently described score for small vascular lesions (27), we rated the total MRI burden of CSVD on an ordinal scale from 0 to 4. Lacunes (1 point if present ≥1 lacune); moderate to severe (grade 2–4) perivascular spaces in the basal ganglia (1 point); periventricular white matter hyperintensities (WMH) Fazekas 3 and/or deep WMH Fazekas 2-3 (1 point); one or more cerebral microbleeds (1 point). Based on the results of the analysis, the patients were divided into three groups: CSVD burden scores of 0, 1, and 2–4. The number of CMB on the MRI SWI sequence was independently determined by two experienced neuroradiologists. CMB was defined as hypointense lesions < 10 mm within the brain parenchyma on SWI images. Characteristic changes similar to CMB, such as calcifications, cerebral venules, cavernous hemangiomas, and malformations, are not included in CMB (28). In the case of disagreement, the number of CMBs was determined by the consensus of two evaluation experts.

### Statistical analysis

SPSS software (version 20.0; SPSS, Chicago, IL, USA), GraphPad Prism 8.0 (GraphPad Software; La Jolla, CA, USA), and 4.0.4 (ggplot2, ggpubr, mediation, and QuantPsyc) were used for the analysis and visualization. To describe demographic data, the Mann–Whitney U test was used for continuous variable intergroup comparisons, such as age, education, and MMSE scores. Chi-square analysis was used for categorical variables, including sex and APOE genotype. AD biofluid biomarker measurements including Aβ42, Aβ40, p-tau, t-tau, Aβ42/Aβ40 ratio, Qalb, and the CSVD burden were compared using the Kruskal–Wallis test, with age, sex, and APOE genotype as covariates.

To explore the relationship between BBB breakdown and CSVD burden, statistical correlations between CSF biomarkers and plasma biomarkers were analyzed by multiple linear regression, adjusted for age, sex, and APOE genotype. The correlation between Qalb and CMB was analyzed by multiple linear regression, adjusted for age, sex, APOE genotype, and Aβ42. To determine the pathological effect of APOE4 in the BBB or CSVD, Mann–Whitney U test was performed to analyze group differences. A *P* < 0.05 was considered statistically significant in all two-sided tests.

## Results

### Characteristics and the core AD biomarkers of participants

A total of 139 patients were selected: 38 individuals with cognitively normal (CN) and 101 clinically diagnosed (^18^F-florbetapir PET positive) patients with AD. The demographic data, core biomarkers, and clinical features were shown in Table 1. Among the participants, the mean age (*p* = 0.09), years of education (*p* = 0.65), and sex ratio (*p* = 0.12) did not differ between the CN and AD groups. As expected, significant differences in the MMSE score (*p* < 0.0001) and APOE4 status (*p* < 0.0001) were observed between these two groups. The mean Qalb value (*p* = 0.0024), CSVD burden (*p* < 0.0001), CMB numbers (*p* = 0.03), and PVS numbers (*p* = 0.0009) in the AD group were significantly higher than those in the CN group, which confirmed severe blood vessel damage and BBB breakdown in AD. Levels of CSF and plasma p-tau181 (*p* < 0.0001) and t-tau (*p* < 0.0001) were both higher in the AD group compared to those in the CN group. On the contrary, except for CSF Aβ40 levels (*p* = 0.03), CSF and plasma Aβ42 (*p* < 0.0001) and Aβ42/Aβ40 ratio (*p* < 0.0001) were significantly lower in the AD group. We also confirmed that CSF was significantly associated with plasma levels of t-tau, p-tau181, and the Aβ42/ Aβ40 ratio in Supplementary Figure S1.

**Table 1:** Demographic characteristics and fluid biomarkers of subjects.

	**CN** **(*n* = 38)**	**AD** **(*n* = 101)**	***p*** **value**
Age (years), mean ± SD	63.2 ± 7.2	64.5 ± 7.5	0.09
Male, n (%)	18 (47.4%)	35 (34.7%)	0.12
Education (years), mean ± SD	8.1 ± 4.7	7.5 ± 4.9	0.65
MMSE, mean ± SD	26.5 ± 3.1	14.9 ± 6.9	<0.0001^*^
APOE4 carriers, n (%)	6 (15.8%)	63 (62.4%)	<0.0001^*^
Qalb, mean ± SD	7.3 ± 3.5	9.4 ± 4.3	0.0024^*^
CSVD burden, median (IQR)	0	1	<0.0001^*^
CMBs (number), median (IQR)	0 (0,0)	0 (0,3)	0.03^*^
PVS (number), median (IQR)	0 (0,0)	0 (0,5)	0.0009^*^
Lacunes (number), median (IQR)	0 (0,2)	0 (0,3)	0.64
CSF Aβ40, mean ± SD	7,641 ± 2,891	6,400 ± 2,836	0.03^*^
CSF Aβ42, mean ± SD	682.4 ± 330.8	314.5 ± 181.7	<0.0001^*^
CSF Aβ42/40, mean ± SD	0.09 ± 0.02	0.05 ± 0.02	<0.0001^*^
CSF T-tau, mean ± SD	83.4 ± 31.2	179.5 ± 121.1	<0.0001^*^
CSF P-tau181, mean ± SD	38.1 ± 15.3	119.3 ± 78.6	<0.0001^*^
Plasma Aβ40, mean ± SD	182.1 ± 27.5	190.2 ± 46.8	0.52
Plasma Aβ42, mean ± SD	12.1 ± 3.2	9.8 ± 3.1	<0.0001^*^
Plasma Aβ42/40, mean ± SD	0.07 ± 0.01	0.05 ± 0.01	<0.0001^*^
Plasma T-tau, mean ± SD	2.4 ± 0.6	3.0 ± 1.1	<0.0001^*^
Plasma P-tau181, mean ± SD	2.4 ± 1.3	5.7 ± 2.7	<0.0001^*^

* Significant difference (p < 0.05) between CN and AD subjects. Aβ40, amyloid beta 40; Aβ42, amyloid beta 42; Qalb, albumin quotient; CMB, cerebral microbleeds; CSF, cerebrospinal fluid; IQR, interquartile range; MMSE, Mini Mental State Examination; P-tau, tau phosphorylated at threonine 181; T-tau, total tau.

Units for CSF and plasma biomarkers are pg/ml.

### BBB (Qalb) and CSVD burden of participants

**Figure 1:**
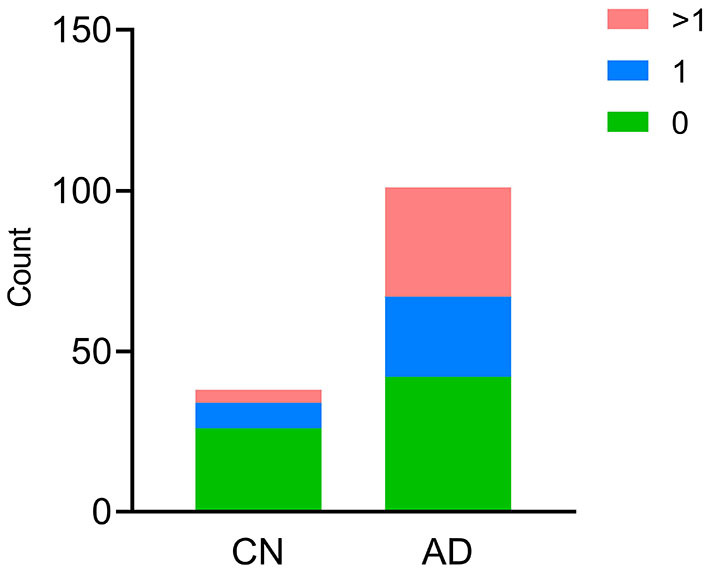
Cerebral small-vessel disease (CSVD) burden in CN and AD individuals. The number of participants with different CSVD burdens is shown, green bar CSVD burden = 0, blue bar CSVD burden = 1, red bar CSVD burden>1.

**Figure 2:**
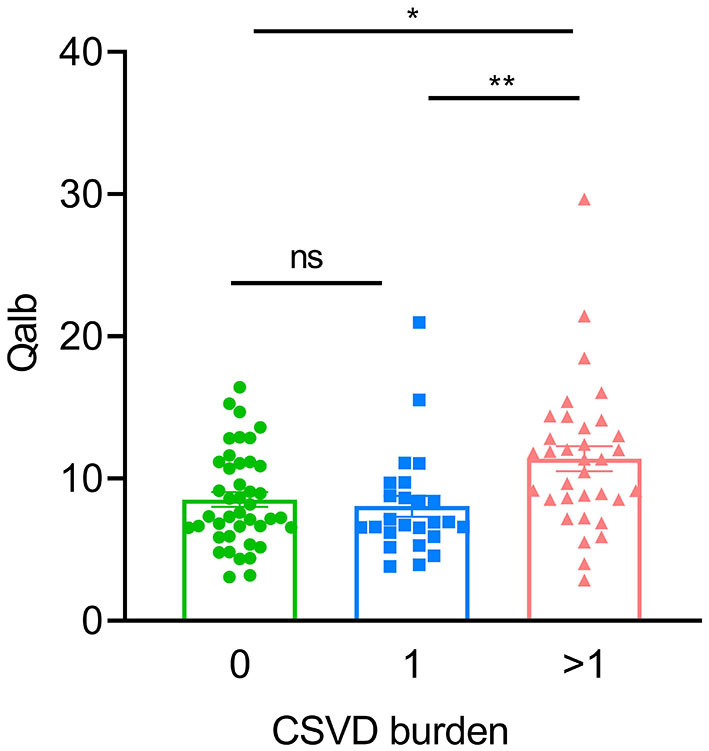
Comparisons of Qalb values in patients with AD with different CSVD burdens. Each dot refers to the value of indicated measures of a single patient with AD. Error bars indicate SEM. *p < 0.05, **p < 0.01; ns, not significant, Kruscal–Wallis test.

**Figure 3:**
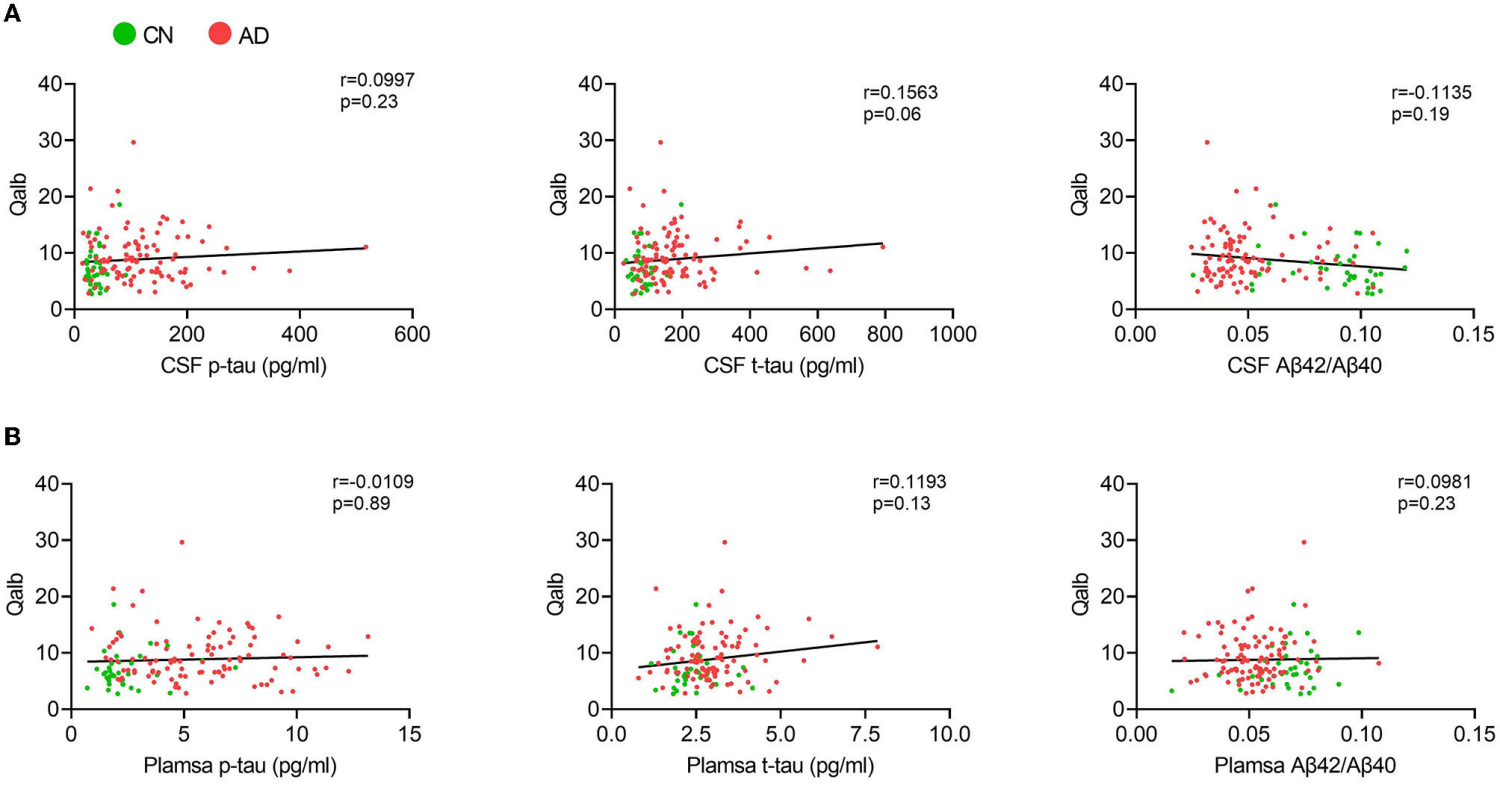
Correlation between Qalb and fluid biomarkers. **(A)** Correlation between CSF biomarkers and Qalb. **(B)** Correlation between plasma biomarkers and Qalb.

In the AD group, the number of patients with CSVD total load scores of 0, 1, and >1 was 42(41.6%), 25 (24.7%), and 34 (33.7%), respectively. In the CN group, there were 26 (68.4%), 8 (21.1%), and 4 (10.5%) patients, respectively (Figure 1). In all individuals, BBB function was significantly reduced with increasing CSVD total load, as indicated by the increased Qalb values. As shown in Figure 2, patients with a CSVD total load >1 had significantly higher Qalb values than those in the other two groups. Meanwhile, Qalb showed a negative correlation with the CSF Aβ42/Aβ40 ratio (*r* = −0.1830, *p* = 0.03) when adjusting for age and sex, but the correlation disappeared when adjusting for age, sex, and APOE genotype (*r* = –0.1135, *p* = 0.19) (Figure 3).

### Associations of CMB with BBB breakdown and fluid biomarkers in patients with AD

**Figure 4:**
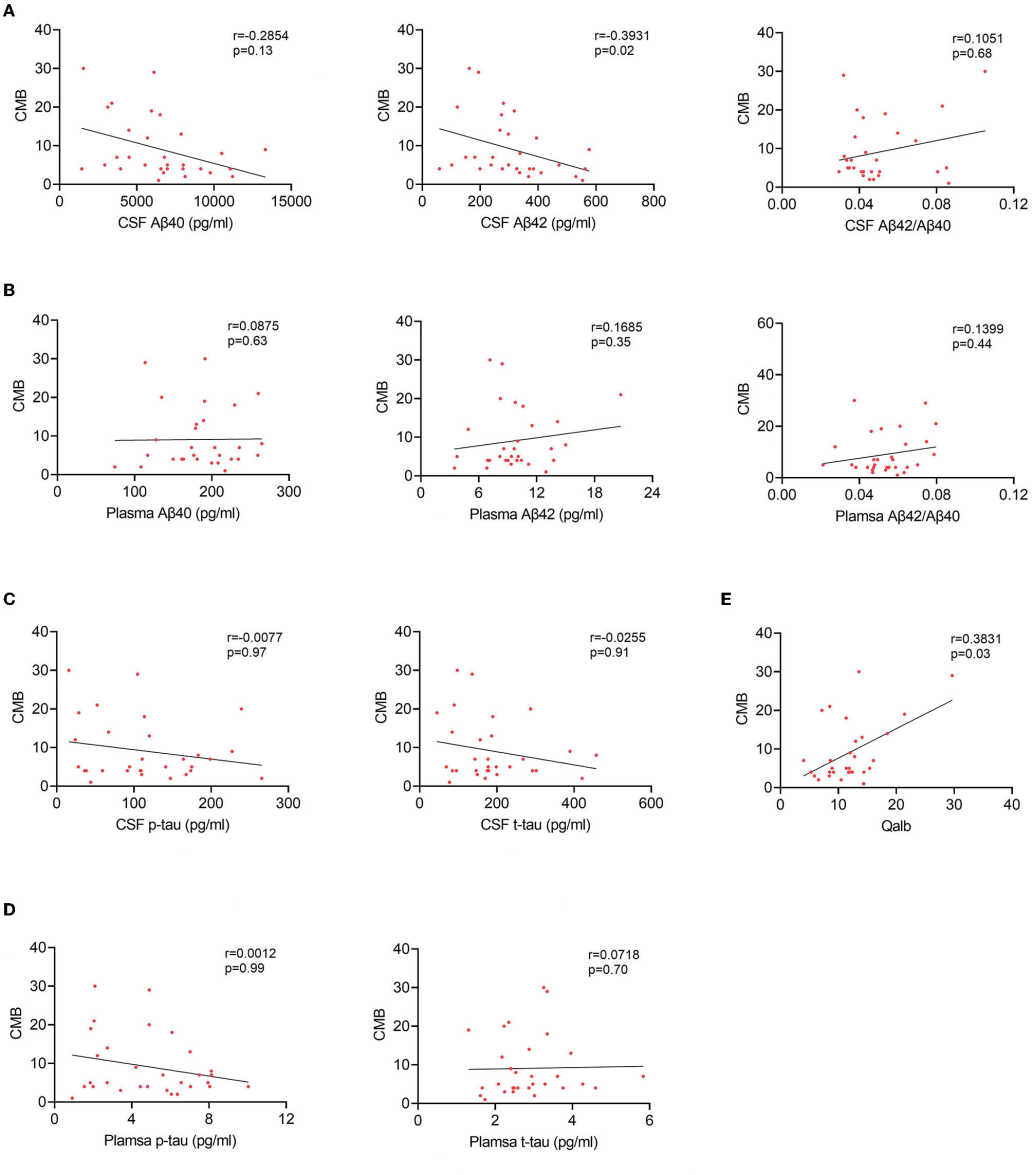
Correlation between CMB and fluid biomarkers in patients with AD with CMB. **(A)** Correlation between CSF Aβ and CMB. **(B)** Correlation between plasma Aβ and CMB. **(C)** Correlation between CSF tau and CMB. **(D)** Correlation between plasma tau and CMB. **(E)** Correlation between Qalb and CMB.

To explore the relationship between BBB breakdown and CMB, and the effects of CMB on AD pathology, patients with AD were divided into two groups according to the presence or absence of CMB. The demographic characteristics are presented in Table 2. As shown, patients with AD who had CMB were older than those without CMB (*p* < 0.0001). In AD with the CMB group, only one case showed simple deep/cerebellar microbleeds, 14 cases with simple lobar microbleeds, and 15 cases with deep mixed lobar microbleeds. Meanwhile, the numbers of lobar microbleeds were significantly higher than the number of deep/cerebellar microbleeds [0, (0, 3); 0, (0, 0) *p* < 0.0001]. Interestingly, the mean Qalb value in patients with CMB was significantly higher than that in patients without CMB (*p* < 0.0001). As for the fluid biomarkers, there was no difference in Aβ, tau concentrations, or Aβ42/Aβ40 ratio between the two groups. We further performed regression analyses to identify the associations between CMB and biomarkers. An increasing number of CMB was associated with a low level of CSF Aβ42 *r* = –0.3931, *p* = 0.02) in patients with AD (Figure 4A). No significant association was found between CMB and other CSF or plasma biomarkers (Figures 4B–D). Notably, CMB was significantly associated with Qalb (*r* = 0.3831, *p* = 0.03) after adjusting for age, sex, APOE genotype, and CSF Aβ42 (Figure 4E).

**Table 2:** Demographic characteristics and fluid biomarkers of AD subjects with or without CMB.

	**AD (CMB-)** **(*n* = 71)**	**AD (CMB+)** **(*n* = 30)**	***p*** **value**
Age (years), mean ± SD	63.3 ± 7.4	67.4 ± 7.0	0.0096^*^
Male, n (%)	26 (36.6%)	9 (30.0%)	0.65
Education (years), mean ± SD	7.5 ± 4.9	7.6 ± 5.0	0.10
MMSE, mean ± SD	15.2 ± 7.3	14.0 ± 5.7	0.43
APOE4 carriers, n (%)	45 (63.4%)	18(60.0%)	0.82
Qalb, mean ± SD	8.3 ± 3.4	11.9 ± 5.1	0.0001^*^
CSVD burden, median (IQR)	0	2	<0.0001^*^
CSF Aβ40, mean ± SD	6,338 ± 2,834	6,544 ± 2,883	0.05
CSF Aβ42, mean ± SD	316.6 ± 198.4	309.5 ± 137.3	0.22
CSF Aβ42/40, mean ± SD	0.05 ± 0.02	0.05 ± 0.02	0.53
CSF T-tau, mean ± SD	175.3 ± 128.0	189.3 ± 104.1	0.07
CSF P-tau181, mean ± SD	121.3 ± 82.8	114.6 ± 68.8	0.40
Plasma Aβ40, mean ± SD	192.8 ± 46.5	184.0 ± 47.6	0.58
Plasma Aβ42, mean ± SD	9.8 ± 2.9	9.8 ± 3.5	0.98
Plasma Aβ42/40, mean ± SD	0.05 ± 0.01	0.05 ± 0.01	0.51
Plasma T-tau, mean ± SD	3.0 ± 1.1	2.9 ± 1.0	0.86
Plasma P-tau181, mean ± SD	6.0 ± 2.8	4.9 ± 2.5	0.56

* Significant difference (p < 0.05) between subjects with and without CMB.

Aβ40, amyloid beta 40; Aβ42, amyloid beta 42; Qalb, albumin quotient; CSF, cerebrospinal fluid; IQR, interquartile range; MMSE, Mini Mental State Examination; P-tau, tau phosphorylated at threonine 181; T-tau, total tau.

Units for CSF and plasma biomarkers are pg/ml.

### Effects of APOE4 on CSVD burden and BBB breakdown

**Figure 5:**
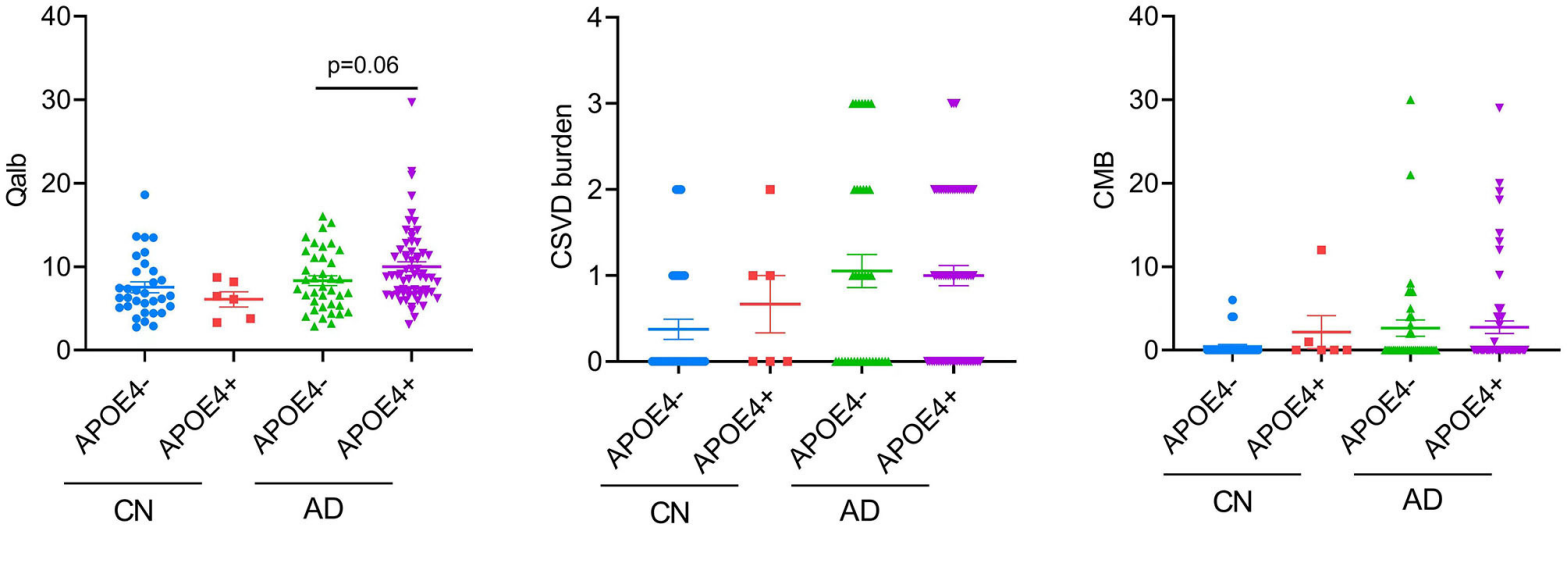
APOE4 effects on BBB function and CSVD severity. Each dot represents a single participant.

Considering the high proportion of APOE4 carriers in patients with AD and the effect of the genotype on the previous results, we analyzed the differences in CSVD load, CMB, and Qalb between APOE4 carriers and non-carriers. Except for the tendency of APOE4 carriers to have higher Qalb values than those of non-carriers in the AD group, no other significant differences were observed (Figure 5).

## Discussion

In this study, we found that the Qalb value and CSVD burden in patients with AD were significantly higher than those in the cognitively normal population, and the Qalb value positively correlated with the CSVD burden. Additionally, we explored the relationship between CMB, an important marker of CSVD, and Qalb in AD. Patients with AD were divided into two groups: those with CMB and those without CMB. The average age of patients with AD with CMB was higher, and they had a higher CSVD burden and APOE4 carrier rate. In the CMB group, Qalb increased with the increasing number of CMB. Additionally, the number of CMB negatively correlated with CSF Aβ42 concentration after adjusting for age, sex, and APOE genotype. Meanwhile, more microbleeds were accompanied by a higher Qalb after adjusting for age, sex, APOE genotype, and CSF Aβ42. This reflects that BBB damage may exacerbate cerebral microbleeds, which is independent of Aβ pathology in AD progression. Finally, we explored the differences in Qalb, CSVD burden, and CMB between APOE4 carriers and non-carriers. Except for the trend of a higher Qalb in patients with AD with APOE4, no other differences were found.

In recent years, the role of BBB damage in AD has been widely explored. Generally, BBB permeability may promote the accumulation of Aβ (29–32). In previous studies, Qalb was confirmed to be associated with the BBB and CSVD burden in AD (33, 34). When the BBB is compromised, the permeability of albumin and IgG into the normal CSF is altered. Protein and other molecules in the blood may enter the brain through the enlarged BBB gap, leading to elevated albumin concentration in CSF. It is generally recognized that higher Qalb represented greater damage to the BBB in neurodegenerative dementias (16, 33). In this study, we found that patients with AD had higher Qalb. Moreover, the possibility of higher Qalb accompanied by increased Aβ deposition was also confirmed. However, the correlation between Qalb and Aβ decreased after adjusting for the APOE genotype, which may indirectly indicate that APOE4 is involved in the destruction of BBB by Aβ pathology. Therefore, we analyzed the differences in BBB function between APOE4 carriers and non-carriers. Although no significant difference was observed between the groups, there was a trend for higher Qalb in APOE4 carriers in the AD group. These results also further confirmed that the genetic polymorphism of APOE may be indirectly involved in the BBB damage in AD, by mediating Aβ pathology.

In addition to BBB damage, cerebrovascular imbalance and vascular factors also contribute to the occurrence and development of AD. In our study, patients in the AD group had a higher CSVD burden. To further explore the possible causal relationship between BBB damage and AD combined with cerebral microangiopathy, we also performed a subgroup comparison of CMB. In our study, CMB number positively correlated with Qalb in patients with AD, which was independent of age, sex, APOE genotype, and Aβ pathology. Vascular injury can lead to a prominent BBB rupture, presenting as cerebral microhemorrhage, which is commonly seen in individuals with an increased genetic risk for AD and MCI. BBB damage is more severe in patients with AD with more microbleeds, which further indicates that the occurrence of CMB is related to BBB dysfunction. Increased BBB permeability leads to the extravasation of red blood cells from small cerebral vessels, which is a major feature of CMB. BBB dysfunction allows neurotoxic blood-derived components, blood cells, and pathogens to enter the brain, leading to the deterioration of the brain environment, further aggravating brain degeneration and CMB occurrence (34). The location of the microbleeds is related to its etiology: CAA causes lobar microbleeds, while hypertensive vascular lesions cause microbleeds commonly in the basal ganglia, thalamus, cerebellum, and brainstem. Microbleeds in patients with AD are mainly observed in the lobars, especially the occipital lobes (35, 36). However, the molecular and cellular mechanisms underlying BBB dysfunction associated with CMB development remain unclear. Previous studies on CMB and AD have found that cerebral microbleeds may be a bridge between other important theoretical hypotheses about the pathogenesis of AD, the amyloid cascade hypothesis (37–40). In our cohort, we found a more severe CSVD burden and CMB in the probable AD population defined as positive for ^18^F-florbetapir PET. Similarly, we also observed a higher proportion of combined cortical microhemorrhage in patients with AD. Although no differences in biomarkers were found in patients with AD with or without CMB in the present study, correlation analysis revealed that CSF Aβ42 levels decreased with an increase in CMB number, which was consistent with a previous study (41). This suggests that the Aβ metabolic pathway may be directly involved in the formation of intracerebral microbleeds in patients with AD. Even after adjustment for Aβ42, the correlation between CMB and Qalb remained significant, which further indicated that BBB damage may directly contribute to the development of CMB in AD.

Because of the limited sample size, we did not distinguish the specific brain lobe and deep regions of CMB to further analyze their relationship between AD pathology and BBB damage. Further studies should include more samples and perform detailed analyses to explore the relationship between BBB and CSVD in AD.

Overall, our study provided evidence that patients with AD have a higher Qalb and CSVD burden, and Qalb value positively correlated with CSVD burden. Furthermore, Qalb was closely associated with CMB when Aβ42 levels were adjusted. This further indicates that BBB damage may be involved in the pathological mechanism of AD and may be a risk factor for CSVD pathogenesis.

## Data availability statement

The raw data supporting the conclusions of this article will be made available by the authors, without undue reservation.

## Ethics statement

The studies involving human participants were reviewed and approved by Department of Ethics Committee, the First Affiliated Hospital of USTC, Division of Life Sciences and Medicine, University of Science and Technology of China, Hefei, China. The patients/participants provided their written informed consent to participate in this study.

## Author contributions

XL, QW, and ZC designed this study, analyzed and interpreted the data, and wrote the manuscript. YW and CL recruited patients and collected samples. PW, MN, and CL performed MRI and PET scanned and analyzed. FG and LD performed the experiments. All authors are accountable for the contents of this research.

## Supplementary material

Supplementary Figure S1 Correlation between CSF and plasma biomarkers.
