# Supplementary figures and images for: Correlation of blood–brain barrier leakage with cerebral small vessel disease including cerebral microbleeds in Alzheimer's disease
Source: Front Neurol. 2023 Feb 16;14:1077860. doi: 10.3389/fneur.2023.1077860 (PMC9978776; doi:10.3389/fneur.2023.1077860)

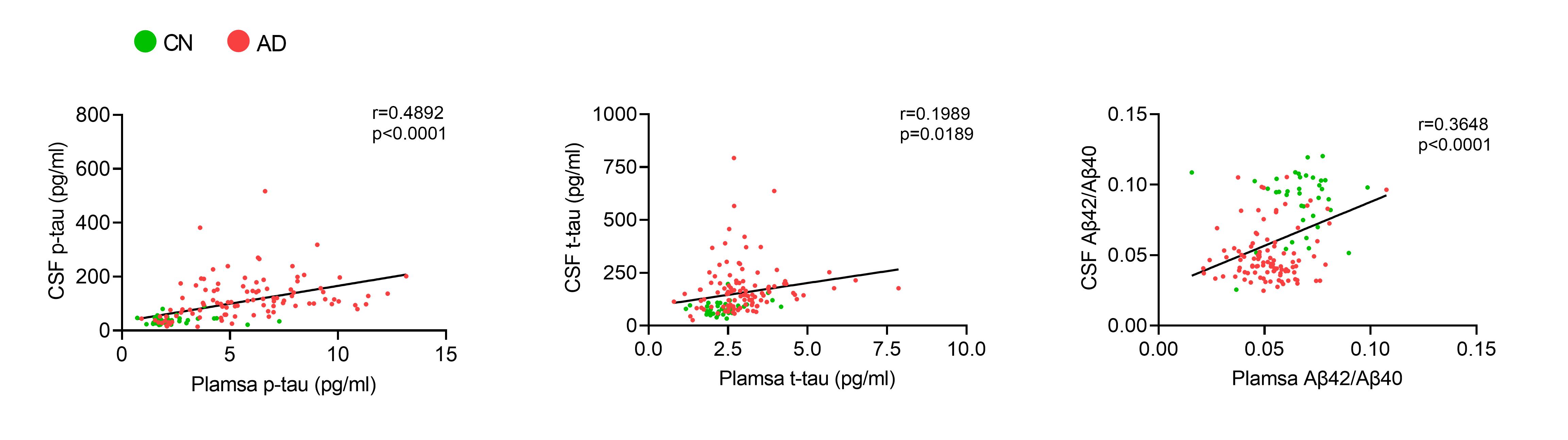

Supplement: Supplementary Figure S1 — Correlation between CSF and plasma biomarkers. [file Image_1.JPEG]
